# Expression of *RMRP* RNA is regulated in chondrocyte hypertrophy and determines chondrogenic differentiation

**DOI:** 10.1038/s41598-017-06809-5

**Published:** 2017-07-25

**Authors:** Mandy M. F. Steinbusch, Marjolein M. J. Caron, Don A. M. Surtel, Franziska Friedrich, Ekkehart Lausch, Ger J. M. Pruijn, Wouter Verhesen, Blanche L. M. Schroen, Lodewijk W. van Rhijn, Bernhard Zabel, Tim J. M. Welting

**Affiliations:** 1 Laboratory for Experimental Orthopedics, Department of Orthopedic Surgery, Maastricht University Medical Centre, Maastricht, The Netherlands; 2 Department of Pediatrics, Medical Center – University of Freiburg, Faculty of Medicine, University of Freiburg, Freiburg, Germany; 3 Department of Biomolecular Chemistry, Institute for Molecules and Materials and Radboud Institute for Molecular Life Sciences, Radboud University, Nijmegen, The Netherlands; 4 Center for Heart Failure Research, Department of Cardiology, CARIM School for Cardiovascular Diseases, Maastricht University, Maastricht, The Netherlands; 5 Medical Faculty, Otto-von-Guericke-University of Magdeburg, Magdeburg, Germany

## Abstract

Mutations in the *RMRP*-gene, encoding the lncRNA component of the RNase MRP complex, are the origin of cartilage-hair hypoplasia. Cartilage-hair hypoplasia is associated with severe dwarfism caused by impaired skeletal development. However, it is not clear why mutations in *RMRP* RNA lead to skeletal dysplasia. Since chondrogenic differentiation of the growth plate is required for development of long bones, we hypothesized that *RMRP* RNA plays a pivotal role in chondrogenic differentiation. Expression of *Rmrp* RNA and RNase MRP protein subunits was detected in the murine growth plate and during the course of chondrogenic differentiation of ATDC5 cultures, where *Rmrp* RNA expression was found to be correlated with chondrocyte hypertrophy. Genetic interference with *Rmrp* RNA expression in ATDC5 cultures caused a deregulation of chondrogenic differentiation, with a prominent impact on hypertrophy and changes in pre-rRNA processing and rRNA levels. Promoter reporter studies showed that *Rmrp* RNA expression responds to chondrogenic morphogens. Chondrogenic trans-differentiation of cartilage-hair hypoplasia fibroblasts was impaired with a pronounced impact on hypertrophic differentiation. Together, our data show that *RMRP* RNA expression is regulated during different stages of chondrogenic differentiation and indicate that *RMRP* RNA may play a pivotal role in chondrocyte hypertrophy, with potential consequences for CHH pathobiology.

## Introduction

RNase MRP is a small nucleolar ribonucleoprotein (snoRNP) particle, consisting of the *RMRP* long non-coding RNA (lncRNA) and several protein subunits (i.e. Rpp14, Rpp20, Rpp21, Rpp25, Rpp30, Rpp38, Rpp40, hPop1, hPop4 and hPop5^1^). RNase MRP is ubiquitously present in eukaryotes and acts as an endoribonuclease which cleaves several RNA substrates. It has been reported to be involved in mitochondrial DNA replication^2^, cleaves pre-rRNA in the internal transcribed spacer 1 (ITS1)^3, 4^, plays a role in cell cycle regulation by cleaving cyclin b2 mRNA^5^, and is able to cleave viperin mRNA^6^. In addition, it has been shown that *RMRP* RNA and the telomerase-associated reverse transcriptase (TERT) protein are able to form a complex that exerts RNA-dependent RNA polymerase activity, which is able to generate a double stranded *RMRP* RNA molecule. Via a Dicer-dependent route siRNAs are generated from this molecule, downregulating cellular *RMRP* levels and providing a negative feedback mechanism controlling *RMRP* RNA levels^7^. In addition, *RMRP* RNA is the source of at least two other short RNAs designated *RMRP*-S1 and *RMRP*-S2, which function as miRNAs and have gene-silencing activity relevant for human cartilage-hair hypoplasia (CHH)^8^. Recently, *RMRP* RNA has been shown to interact with DDX5 and play a role in RORγt-dependent TH17 biology^9^. RNase MRP is structurally and functionally related to the RNase P endoribonuclease, which has a unique RNA component, but shares many protein subunits with RNase MRP^10^. RNase P is critically involved in maturation of tRNAs by cleaving the 5′ leader of pre-tRNA and is involved in RNA polymerase I and III transcriptional activity^11–13^.

Mutations in the *RMRP* gene are the cause of a severe form of dwarfism known as the cartilage-hair hypoplasia^14^ (CHH: OMIM #250250) – anauxetic dysplasia^15^ (AAD: OMIM #607095) spectrum of disorders^16^. CHH is also termed metaphyseal chondrodysplasia, McKusick-type^17^. To date, more than hundred individual CHH-pathogenic mutations have been identified in the *RMRP* gene^14^. These mutations are located either in the transcribed region or in the proximal promoter region, often between the TATA box and the transcription start site. Promoter mutations may result in inefficient transcription of the *RMRP* gene, whereas mutations in the transcribed region are thought to influence RNase MRP complex assembly, enzymatic activity, subcellular localization, substrate recognition or RNA stability^14^.

One phenotypic hallmark of CHH is short-limbed dwarfism accompanied by abnormal growth plate development. Other symptoms include sparse thin hair, anaemia, Hirschsprung’s disease, bronchiectasis, and impaired T-cell immunity. In addition, adult patients have a predisposition to certain cancers (i.e. squamous cell carcinoma, basal cell carcinoma and non-Hodgkin lymphoma)^16, 18^. Longitudinal bone growth during skeletal development and lengthening of the limbs depends on endochondral ossification. Endochondral ossification is a multistage process in the growth plates of developing long bones. Growth plates are populated by highly proliferative chondrocytes, which differentiate into mineralizing hypertrophic chondrocytes that either die from apoptosis or transdifferentiate into osteoblasts^19–21^. The remaining mineralized extracellular matrix (ECM) provides a scaffold for osteoblasts and osteoclasts to adhere and remodel, setting the stage for bone apposition and thus longitudinal bone growth and limb development^22, 23^. In the proliferative zone of the growth plate, Sox9 drives the expression of type II collagen (Col2a1), the main marker for this stage of chondrogenic differentiation. Runx2 and Mef2c drive the expression of type X collagen (Col10a1) during the hypertrophic phase of chondrogenic differentiation. Many skeletal abnormalities have been described that are often caused by mutations in genes involved in growth plate development^24^.

Although the involvement of the *RMRP* RNA in the molecular pathology of CHH has been recognized, its role in growth plate biology remains elusive. As abnormal growth plate development is one of the radiological characteristics of CHH^25^, and chondrogenic differentiation in the growth plates is required for skeletal development^23^, we hypothesized that the *RMRP* lncRNA may also function as an important regulator of chondrogenic differentiation.

## Materials and Methods

### *In situ* hybridization (ISH)

Decalcified knee epihyseal growth plates of 6-week-old C57BL/6 mice (use of mouse growth plates was approved by the Maastricht University Animal Ethics Committee, according to Dutch law; and methods utilized to obtain growth plates were carried out in accordance with Maastricht University Animal Ethics Committee and Dutch law) were embedded in paraffin and 5 μm sections were cut. Sections were deparaffinized in a xylene/ethanol series ending in PBS (136 mM NaCl (Merck Millipore, Darmstadt, Germany), 2.7 mM KCl (Merck Millipore), 9.0 mM Na_2_PO_4_.H_2_O (Merck Millipore), 1.8 mM KH_2_PO_4_ (Merck Millipore)). Antigen retrieval was performed using 20 μg/ml proteinase K (Exiqon, Vedbæk, Denmark). Slides were washed extensively with demineralized water to remove proteinase K. Slides were pre-hybridized with microRNA ISH buffer (Exiqon; miRCURY LNA microRNA ISH optimization kit FFPE). Hybridization was performed for 1 hour at 50 °C on a heating plate using 80 nM of Rmrp RNA double-digoxigenin (DIG)-labeled miRCURY LNA probe (5′DIG-CTGACGGATGACGC-3′DIG; custom ordered at Exiqon) or a double-DIG labeled scrambled LNA miRNA probe (5′-GTGTAACACGTCTATACGCCCA-3′; Exiqon) in miRNA ISH buffer. Slides were washed three times with 5x SSC (0.75 M NaCl, 0.075 M sodium citrate) for 10 minutes at 50 °C. Subsequently slides were blocked with blocking solution (DIG Wash and Block buffer set; Roche, Basel, Switzerland) for 30 minutes and incubated with anti-DIG-Alkaline Phosphatase (AP) Fab fragments (1:500; Roche) in blocking solution for 1 hour. Endogenous AP was blocked with Levamisole solution (Vector Laboratories, Burlingame, CA, USA) and bound anti-DIG-AP Fab fragments were detected with AP substrate (NBT/BCIP; Roche) in demineralized water. Slides were washed in PBS-T (0.1% Tween 20; Sigma-Aldrich, St. Louis, MO, USA), counter-stained with 0.01% FastGreen (Sigma-Aldrich), dehydrated and mounted with Entellan (Merck Millipore).

### Immunohistochemistry (IHC)

Knee joint epiphyseal growth plates of 6-week-old C57BL/6 mice were formalin-fixed and decalcified using 0.5 M ethylenediaminetetraacetic acid (EDTA; VWR Prolabo, Amsterdam, the Netherlands), pH 7.8. After paraffin-embedding 5 μm tissue sections were cut. Sections were deparaffinized in a xylene/ethanol series ending in PBS. For antigen retrieval (COL10A1 detection) sections were treated with 0.4% hyaluronidase (Sigma-Aldrich) at 37 °C for 30 minutes. Sections for detection of RNase MRP protein subunits were incubated in hot citrate buffer (1.8 mM citric acid (Sigma-Aldrich) and 8.2 mM tri-sodium citrate (VWR Prolabo)) for 30 minutes. Endogenous peroxidase activity was inactivated using peroxidase-blocking solution (Dako, Troy, MI, USA). Next, slides were blocked with 10% normal sheep serum in PBS-T. Primary antibodies were incubated for 1 hour at room temperature. Rabbit polyclonal anti-COL10A1^26^ was used at a 1:1,000 dilution. Rabbit polyclonal anti-Rpp25^27^, anti-Rpp30^28^, anti-Rpp38^28^, anti-Rpp40^29^ and anti-hPop1^30^ were used at a 1:100 dilution. Bound antibodies were detected with horseradish peroxidase (HRP)-conjugated anti-rabbit secondary antibodies (Dako EnVision) for 30 minutes at room temperature. DAB chromogen substrate (Dako) was used for detection. Sections were counterstained with haematoxylin (Dako), dehydrated and mounted with histomount (Thermo Shandon, Waltham, MA, USA). Microscopic images were acquired using a Zeiss Axioscope A1.

### Cell culture and differentiation of ATDC5 and MCT cells and siRNA transfection

ATDC5 cells^31^ were cultured in a humidified atmosphere at 37 °C, 5% CO_2_ in proliferation medium (DMEM/F12 (Invitrogen, Carlsbad, CA, USA), 5% FCS (PAA, Pasching, Austria), 1% antibiotic/antimycotic (Invitrogen), 1% NEAA (Invitrogen)). To induce chondrogenic differentiation, cells were plated at 6,400 cells/cm^2^. After 24 hours chondrogenic differentiation was initiated by changing the medium to differentiation medium (proliferation medium supplemented with 10 μg/ml insulin (Sigma-Aldrich), 10 μg/ml transferrin (Roche), 30 nM sodium selenite (Sigma-Aldrich)). Differentiation medium was refreshed every two days for the first 10 days, and each day after day 10. Chondrogenically differentiating ATDC5 cells were transfected (Icafectin; Eurogentec, Seraing, Belgium) with 100 nM siRNA duplexes (custom synthesized by Eurogentec, see Table 1) targeting *Sox9*, *Bapx1* or *Rmrp* RNA on day -1, 2, and 5 during differentiation in the presence or absence of 30 nM BMP-2 (Life Technologies, Carlsbad, CA, USA). Scrambled siRNA duplex was purchased from Eurogentec (REF: SR-CL000–005). Cells were harvested for RNA isolation on day 0, 7 and 10 during differentiation (TRIzol reagent; Invitrogen) and samples were kept at −80 °C until further analysis. In addition, ATDC5 cells were differentiated for 14 days and exposed to 5 or 50 nM PTHrP (Bachem, Bubendorf, Switzerland) from day 10 onwards (medium with PTHrP was refreshed on day 12 during chondrogenic differentiation). MCT cells^32^ were cultured in a humidified atmosphere at the permissive temperature of 32 °C, 8% CO_2_ in proliferation medium (DMEM/F12 (Invitrogen), 10% FCS (PAA), 1% antibiotic/antimycotic (Invitrogen), 1% NEAA (Invitrogen)). Hypertrophic differentiation was induced in the absence or presence of 50 nM PTHrP (Bachem) by switching culture conditions to 37 °C, 5% CO_2_ for 24 hours. Cells were harvested for RNA isolation (TRIzol reagent; Invitrogen) and samples were kept at −80 °C until further analysis. To maintain *Rmrp* knockdown during an extended period of time during chondrogenic differentiation, scrambled (Eurogentec SR-CL000-005) and *Rmrp* RNA (Table 1) siRNAs were either transfected on day 2 and 5 of ATDC5 chondrogenic differentiation for read-out at day 7 or on day 2, 5 and 8 in chondrogenic differentiation for read-out at day 14.

**Table 1:** siRNA sequences.

*siRNA*	*Sense*	*Anti-sense*
siSox9	GACUCACAUCUCUCCUAAUTT	AUUAGGAGAGAUGUGAGUCTT
siBapx1	CAGAGACGCAAGUGAAGAUTT	AUCUUCACUUGCGUCUCUGTT
siRmrp RNA	CAUGUUCCUUAUCCUUUCGTT	CGAAAGGAUAAGGAACAUGTT

### Description of plasmids, transfection and bioluminescence detection

Plasmid constructs were generated using traditional cloning methods and cloned inserts were verified by DNA sequencing. The mouse 1500 nucleotide sequence upstream of the mouse Rmrp transcription start site was amplified from ATDC5 genomic DNA and cloned into the pGluc-Basic vector (NEB) using primer-introduced restriction sites *EcoRV* and *HindIII* (pGluc-Rmrp-prom). As a transfection control the pGL4.20[luc2/Puro] (Promega, Southampton, UK) vector was used in which the CMV promoter was cloned (pGL4.20[luc2/Puro]-CMV). Day 5-differentiating ATDC5 cells in 12 well plates were transfected with 500 ng pGluc-Rmrp-prom and 125 ng pGL4.20[luc2/Puro]-CMV using 1 μg/μl polyethylenimine (PEI; Polysciences, Warrington, PA, USA) transfection reagent. DNA and PEI were complexed for 10 minutes at room temperature in DMEM/F12 (1.9 μl PEI per 625 ng construct per well) and added to the ATDC5 cells. Five hours post-transfection medium was supplemented with BMP-2 (30 ng/ml; Life Technologies), PTHrP (100 nM; Bachem), TGF-β3 (10 ng/ml; Life Technologies), dorsomorphin (10 μM; Santa Cruz Biotechnology, Dallas, TX, USA), (5*Z*)-7-Oxozeaenol (0.5 μM; Merck Millipore), SHH (150 ng/ml; R&D systems, Minneapolis, MN, USA), IHH (150 ng/ml; R&D systems), WNT5A (15 ng/ml; R&D systems), WNT3A (15 ng/ml; Peprotech, Rocky Hill, NJ, USA), GDF-5 (100 ng/ml; Peprotech) and bFGF (15 ng/ml; Peprotech). After 36 hours samples were harvested for bioluminescence detection using the Dual Luciferase reporter assay system (Promega) as described by the manufacturer and measured on a Fluostar Omega plate reader (BMG Labtech, Ortenberg, Germany).

### RT-qPCR

RNA was isolated from TRIzol samples by collecting the aqueous phase after phase separation. RNA was precipitated with isopropanol (30 minutes, −80 °C) and centrifuged for 30 minutes at 20,000 × g, 4 °C. RNA pellets were washed with 80% ethanol and potential DNA contamination was removed by DNase I (Roche) treatment (1 hour, 37 °C). After subsequent ethanol precipitation, RNA was dissolved in 15 μL DNase/RNase free water (Eurogentec). RNA quantity and purity were determined spectrophotometrically (Biodrop, Isogen Life Sciences, Utrecht, the Netherlands). DNA-free total RNA was reverse transcribed using standard procedures and random hexamer priming as described previously^33^. Real time quantitative PCR (RT-qPCR) was performed in 96-well optical plates. For each cDNA sample a mix was prepared consisting of Mesagreen qPCR Mastermix Plus for SYBR Green (Eurogentec) and 300 nM forward and reverse oligonucleotides. Serially diluted standard curves were utilized to quantify gene expression in the samples. A Biorad CFX96 Real-Time PCR Detection System was used for amplification using the following protocol: denaturation at 95 °C for 10 minutes, followed by 50 cycles of amplification (15 seconds 95 °C and 1 minute 60 °C) followed by a dissociation curve. Data were analyzed using Biorad CFX Manager Software version 3.1, based on the relative quantification of mRNA expression of the target gene normalized to a housekeeping gene (ATDC5/MCT: β-actin, human fibroblasts: GAPDH). Primer sequences are depicted in Table 2.

**Table 2:** RT-qPCR primer sequences.

*Gene*	*Forward*	*Reverse*
β-Actin	CCGAGCGCGAGATCGT	TGGCCATCTCGTTCTCGAA
Aggrecan	CATGAGAGAGGCGAATGGAA	TGATCTCGTAGCGATCTTTCTTCT
Alpl (*Mm*)	CCGATGGCACACCTGCTT	GGAGGCATACGCCATCACAT
*ALPL* (*Hs*)	CCGTGGCAACTCTATCTTTGG	CAGGCCCATTGCCATACAG
Bapx1	ACCTGGCAGCTTCGCTGAA	AGGTCGGCGGCCATCT
Col2a1 (*Mm*)	TGGGTGTTCTATTTATTTATTGTCTTCCT	GCGTTGGACTCACACCAGTTAGT
*COL2A1* (*Hs*)	TGGGTGTTCTATTTATTTATTGTCTTCCT	GCGTTGGACTCACACCAGTTAGT
Col10a1 (*Mm*)	CATGCCTGATGGCTTCATAAA	AAGCAGACACGGGCATACCT
*COL10A1* (*Hs*)	ATGATGAATACACCAAAGGCTACCT	ACGCACACCTGGTCATTTTCTG
Clb2	TGCCAAGCTTTCTCTGATGCT	GGGTTCTCCCTGTCCTCGTT
*GAPDH* (*Hs*)	ACTTTGTGAAGCTCATTTCCTGGTA	GTGGTTTGAGGGCTCTTACTCCTT
ITS1 (*Mm*)	TGGGGGGGTGGATGTCTGGAG	CGAGTGATCCACCGCTAAGAGTCGTA
*ITS1* (*Hs*)	TGTGAAACCTTCCGACCCCTCT	CGAGTGATCCACCGCTAAGAGTCGTA
Mef2c	GGGCCTCAATGGCTGTGA	CTCAGACTCAGGGCTGTGACCTA
Osteocalcin	GCGGCCCTGAGTCTGACA	GCCGGAGTCTGTTCACTACCTT
Pop4	GGACACGCAGCCACAGATG	TGACTGAAATAATAGCACCATGAAGAT
Pthrp (*Mm*)	GTTCAGCAGTGGAGTGTC	GATGGTGGAGGAAGAAACG
*PTHrP* (*Hs*)	AAGGGCAAGTCCATCCAAGA	CTCGGCGGTGTGTGGATTTC
Rmrp (*Mm*)	ATACGAGGGACATGTTCCTTATCC	TTGGCGGGCTAACAGTGACT
*RMRP* (*Hs*)	GAGAGTGCCACGTGCATACG	ACGCTTCTTGGCGGACTTT
Rpp30	TCAAAAGACCCCCTGTTAATGTG	GATAGCAGGACCATAGACAAGTTCAA
Rpp40	TGTGTCACTACTTCGATGAACCAA	TGTCTGCAAAGCCTTGAACTGT
Runx2	GACGAGGCAAGAGTTTCACC	GGACCGTCCACTGTCACTTT
5.8S rRNA	CACTCGGCTCGTGCGTCGAT	CGCTCAGACAGGCGTAGCCC
18S rRNA	AGTCCCTGCCCTTTGTACACA	GATCCGAGGGCCTCACTAAAC
28S rRNA	GCCATGGTAATCCTGCTCAGTAC	GCTCCTCAGCCAAGCACATAC
Sox9	AGTACCCGCACCTGCACAAC	TACTTGTAGTCCGGGTGGTCTTTC
Viperin	TGCTATCTCCTGCGACAGCTT	CCTTGACCACGGCCAATC

When not specified, primer sequences were designed for *Mus musculus*. In table *Mus musculus* is abbreviated as *Mm* and *Homo sapiens* as *Hs*.

### Cell staining protocols and assays

#### Crystal Violet, Alcian Blue and Alizarin Red S staining and quantification

Cells were washed two times with 0.9% NaCl and fixed with 4% paraformaldehyde in PBS for 10 minutes at room temperature. Fixed cells were washed 6 times with distilled water and air-dried. Dried fixed cells were incubated for 30 minutes at room temperature with either 0.1% (m/v) Crystal Violet (Sigma-Aldrich); 1% (m/v) Alcian Blue (Acros Organics, Geel, Belgium) in 0.1 M HCl; or 40 mM Alizarin Red S (Sigma-Aldrich), pH 4.2. Cells were washed six times with distilled water to remove unbound stain and allowed to air dry. Pictures were acquired with an Epson V370 flatbed scanner (Epson, Nagano, Japan). Crystal Violet was extracted by incubation with 10% acetic acid (VWR) for 15 minutes on a plate shaker (IKA HS 260 Basic, IKA, Staufen, Germany). Alcian Blue was extracted by incubation with 6 M Guanidine-HCl (Sigma-Aldrich) for 2 hours on a plate shaker (IKA). Alizarin Red S extraction was initiated by incubation with 10% acetic acid (VWR Prolabo) for 30 minutes at room temperature on a plate shaker (IKA). Cells and eluate were further collected using a cell scraper and transferred to a microcentrifuge tube. Samples were heated at 85 °C for 10 minutes and subsequently centrifuged at 20,000 × g for 15 minutes at 4 °C. Ammonium hydroxide (VWR) was added to neutralize the acetic acid until a pH of 4.4 was reached. Extracted Crystal Violet, Alcian Blue and Alizarin Red S were quantified spectrophotometrically at 590 nm, 645 nm or 405 nm (respectively) using a plate reader (ThermoScientific Multiskan FC, Waltham, MA, USA).

#### ALP activity assay

Enzymatic activity of Alkaline Phosphatase in ATDC5 cultures was determined in a colorimetric assay. Cells were lysed in lysis buffer (1.5 M Tris-HCl, pH 9.0; 2% v/v Triton X-100) and homogenized on ice by means of sonication (MSE Soniprep 150, Gemini, Apeldoorn, the Netherlands). Insoluble material was removed by centrifugation (5 minutes, 13,000 at 4 °C). Total protein concentration was determined with a BCA assay (Sigma-Aldrich). In flat-bottom 96 wells plates containing assay buffer (1.5 M Tris-HCl, pH 9.0, 1 mM MgCl_2_; 7.5 mM p-nitrophenyl phosphate), ALP activity was determined by measuring ALP-dependent enzymatic conversion of p-nitrophenyl phosphate to nitrophenyl phosphate by spectrophotometric analysis at 405 nM. A calibration curve containing an increasing concentration of nitrophenyl phosphate was used to determine the absolute amount of ALP-generated nitrophenyl phosphate over time. Values were normalized to total protein concentration and ALP activity expressed as µmol nitrophenyl phosphate/gram/minute.

### Chondrogenic trans-differentiation of human dermal fibroblasts

Ethical permission for the use of dermal fibroblasts was obtained from the medical ethical Institutional Review Board of Freiburg University Hospital and methods and experimental protocols to obtain dermal fibroblasts were carried out in accordance with the Freiburg University Hospital medical ethical Institutional Review Board, according to German law. Informed consent was obtained from all subjects. Human dermal fibroblasts (passage 7–9) were hyperconfluently plated (100,000 cells/cm^2^) in Aggrecan-coated (Sigma-Aldrich; 2.5 μg/cm^2^) wells as previously described^34^. Cells were directly plated in trans-differentiation medium which consisted of DMEM/F12 + Glutamax (Invitrogen), 10% FCS (PAA), 1% antibiotic/antimycotic (Invitrogen), 1% NEAA (Invitrogen) + 1% ITS (Insulin, Transferrin, Selenium-Sodium Pyruvate; Life Technologies), 50 μg/ml ascorbic acid 2-phosphate (Sigma-Aldrich) and 1 ng/ml human recombinant TGF-β3 (Life Technologies). Trans-differentiation medium was refreshed every other day and transdifferentiated cartilaginous nodules were harvested in TRIzol on day 3, 5 and 7 during trans-differentiation. At plating, fibroblasts were harvested for day 0 samples as well.

## Results

### Rmrp RNA and RNase MRP protein subunits are differentially expressed in the developing growth plate

**Figure 1:**
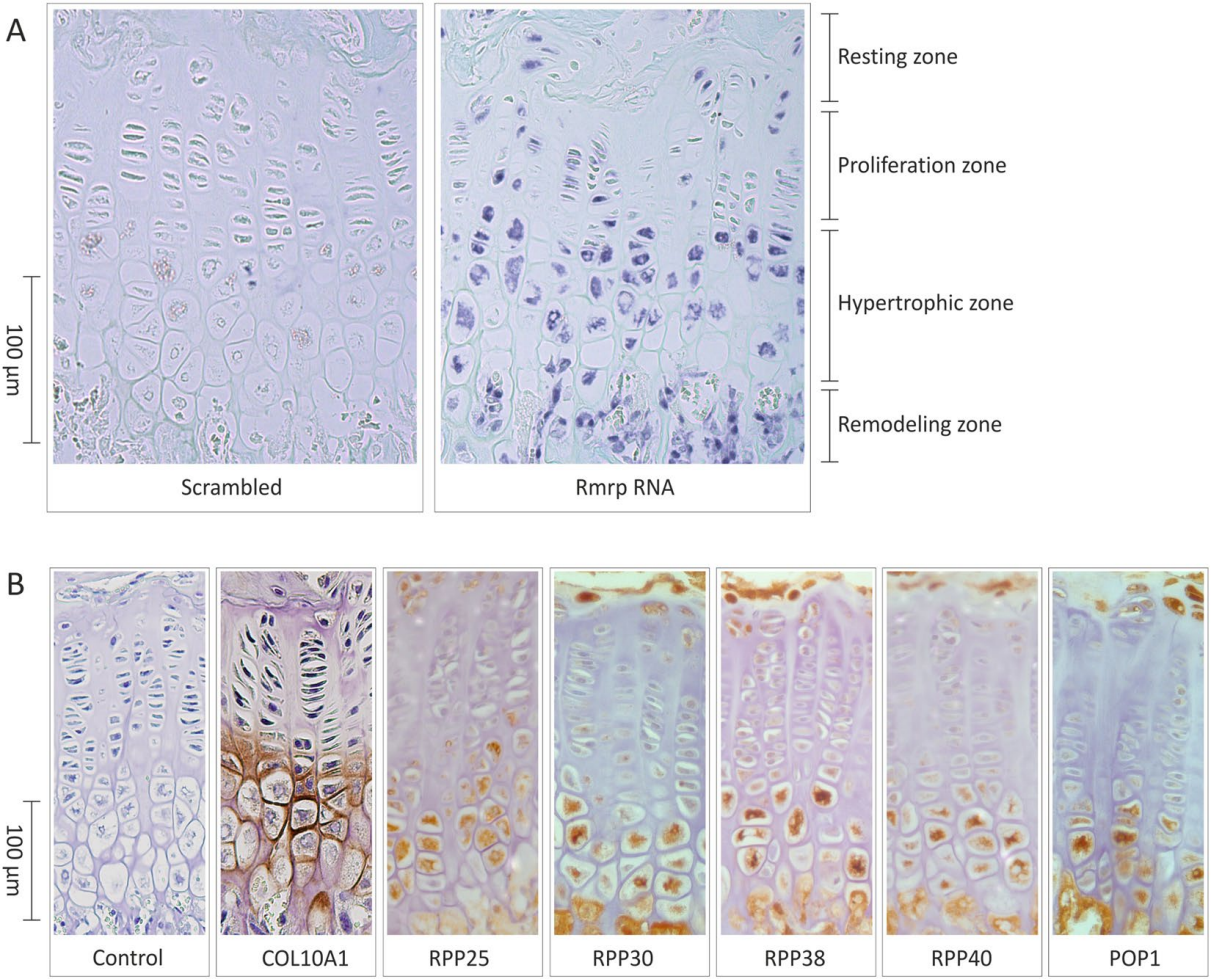
Spatiotemporal expression of *Rmrp* RNA and RNase MRP subunits in the growth plate. Five micrometer-thick formalin-fixed paraffin-embedded tissue sections were prepared from knee joint epiphyseal growth plates of 6-weeks-old mice. (**A**) Expression of *Rmrp* RNA was detected by *in situ* hybridization. A scrambled probe was used as control. (**B**) The expression of COL10A1 and RNase MRP protein subunits RPP25, RPP30, RPP38, RPP40 and POP1 was detected immunohistochemically. Figure shows representative images of the ISH (**A**) IHC (**B**) results from three individual experiments each.

To investigate *RMRP* RNA expression during chondrogenic differentiation of the growth plate, 5 μm tissue sections were prepared from growth plates of 6 weeks-old mice. Expression of *Rmrp* RNA was determined by *in situ* hybridization (Fig. 1A). Resting zone chondrocytes express *Rmrp* RNA and weak expression was observed in the chondrocytes of the proliferative zone. The highest expression levels of *Rmrp* RNA were detected in the hypertrophic zone. Cells in the remodeling zone of the growth plate (osteoclasts and osteoblasts) were also positive for the expression of *Rmrp* RNA. Subsequently, we evaluated the spatiotemporal expression of a number of RNase MRP protein subunits RPP25, RPP30, RPP38, RPP40 and POP1 (Fig. 1B). Expression of COL10A1 was immunohistochemically detected to visualize the location of the hypertrophic zone (Fig. 1B). Without exception, all RNase MRP protein subunits tested displayed a spatiotemporal growth plate distribution pattern highly similar to *Rmrp* RNA; resting zone chondrocytes express RNase MRP subunits, whereas weak expression was observed in chondrocytes of the proliferative zone. Highest RNase MRP protein subunit expression levels were detected in the hypertrophic zone of the developing growth plate.

### Rmrp RNA expression is induced during ATDC5 chondrogenic differentiation

**Figure 2:**
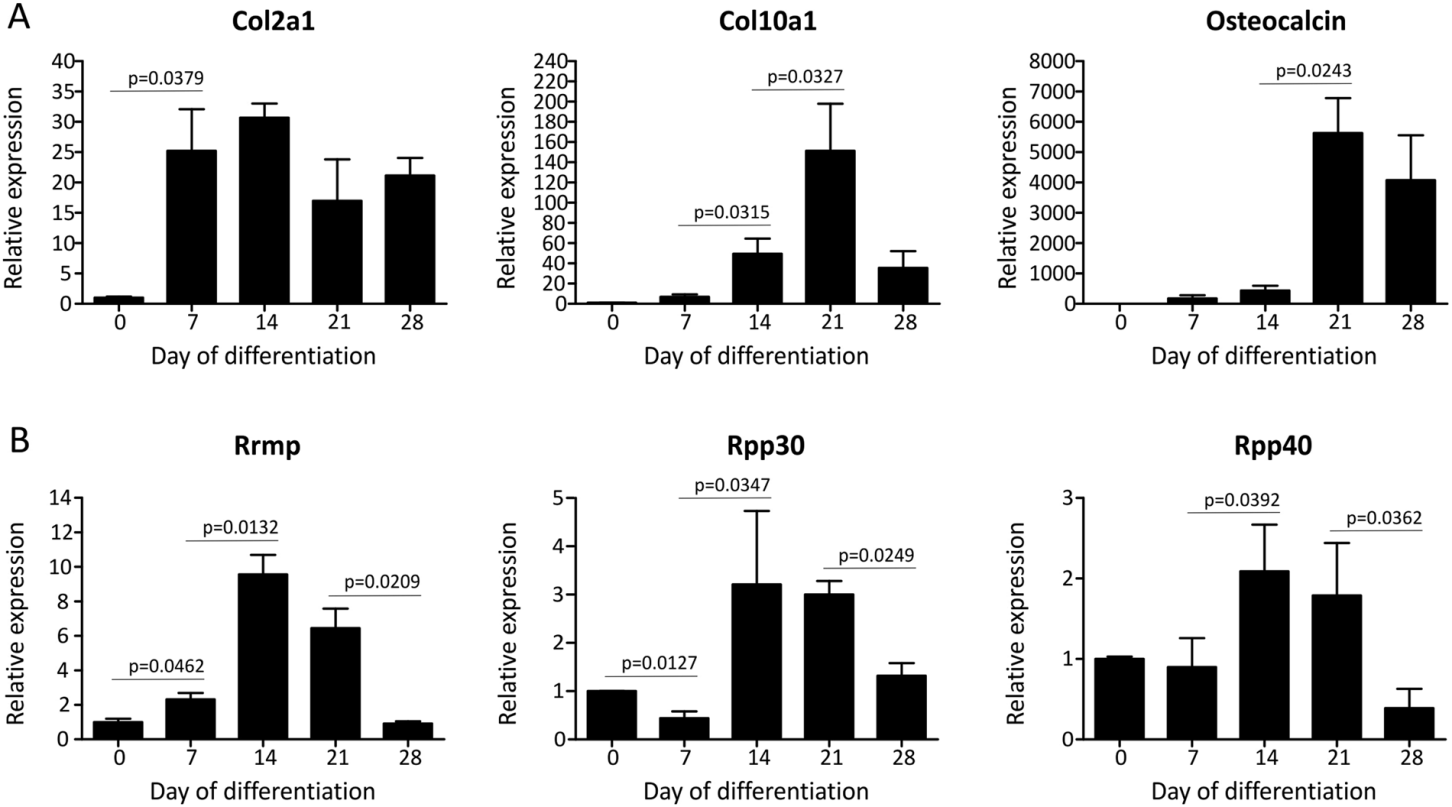
*Rmrp* RNA and RNase MRP subunit expression responds to chondrogenic differentiation of ATDC5 cells. ATDC5 cells were differentiated in the chondrogenic lineage for 0, 7, 14, 21 or 28 days. (**A**) Different stages of chondrogenic differentiation were confirmed by measuring gene expression of *Col2a1*, *Col10a1* and *osteocalcin*. (**B**) Expression of *Rmrp* RNA and RNase MRP protein subunits *Rpp30* and *Rpp40* is depicted as fold induction relative to t = 0. Data was normalized to *β-actin* and represents the average value of 3 biological replicates plus standard deviation. For statistical evaluation an independent samples t-test was performed between each consecutive time point using GraphPad Prism 5. p-values are indicated. Presented graphs are representative examples of three independent experiments.

To study *RMRP* RNA expression in a model for chondrogenic differentiation we used the ATDC5 cell line^31^. ATDC5 cells follow a defined chondrogenic differentiation program after stimulation with insulin, transferrin and sodium selenite. The induction of *Col2a1* expression at day 7 during differentiation marks chondrogenic differentiation. At 14–21 days during differentiation cells have acquired a predominant hypertrophic/mineralizing phenotype as indicated by co-expression of *Col10a1*, and at day 21 mineralization becomes evident by expression of osteocalcin (Fig. 2A). Expression of *Rmrp* RNA was slightly upregulated at day 7 in chondrogenic differentiation, increased further at day 14 in differentiation and showed highest levels at day 14 and 21 in differentiation, predominantly coinciding with peak *Col10a1* expression. *Rmrp* RNA expression decreased again at day 28 during differentiation (Fig. 2B). Expression of RNase MRP protein subunits *Rpp30* and *Rpp40* showed a similar pattern with the highest levels of expression at day 14 and 21 during differentiation (Fig. 2B). Together, these data show that the expression of *Rmrp* RNA responds to induction of chondrogenic differentiation with highest expression levels late in the differentiation program.

### Rmrp expression is positively correlated with the hypertrophic phenotype of the chondrocyte

**Figure 3:**
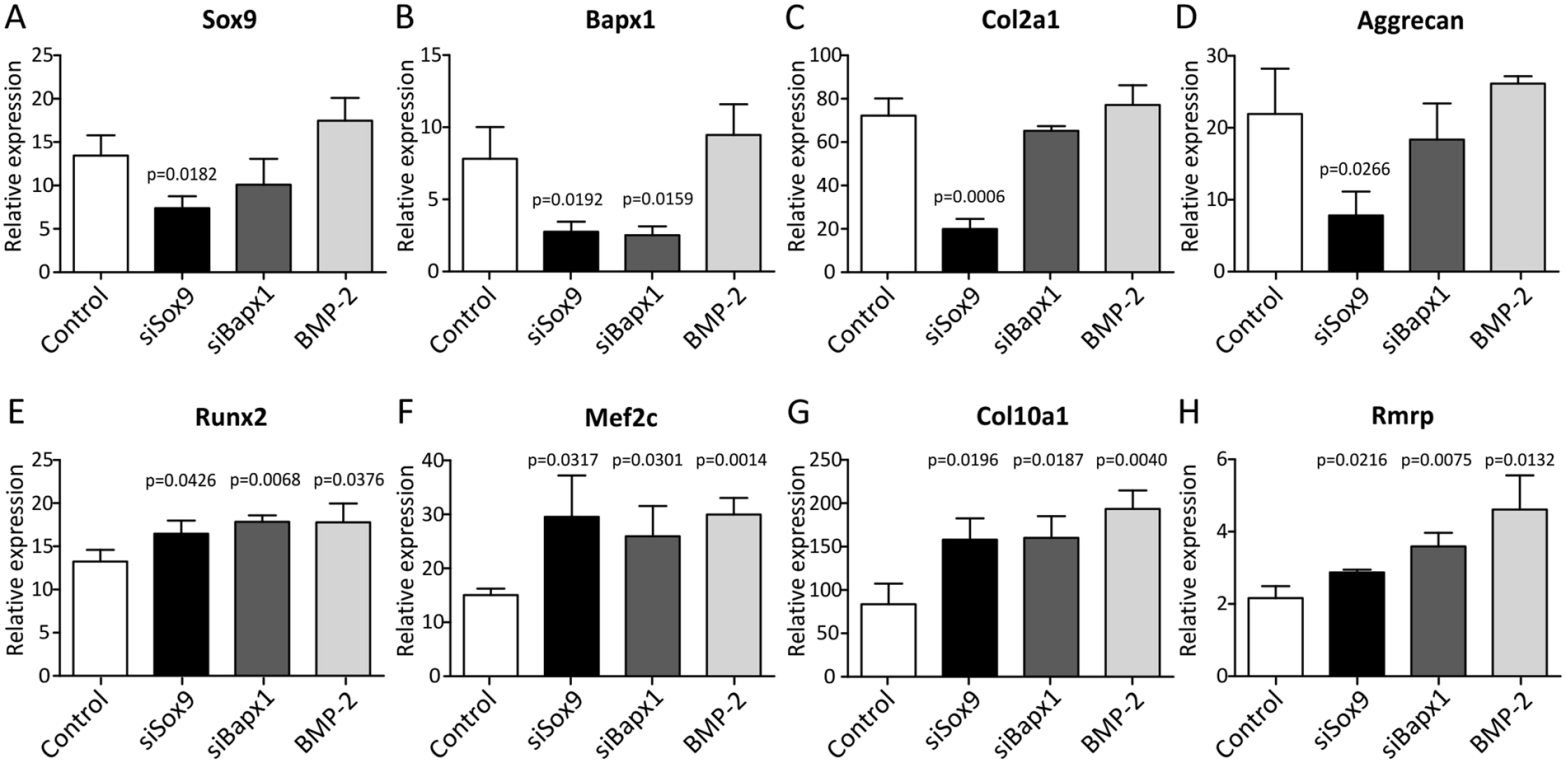
*Rmrp* RNA expression increases with ATDC5 hypertrophic differentiation. ATDC5 cells were transfected with 100 nM siRNA duplexes targeting *Sox9* or *Bapx1* gene expression on day -1, day 2, and day 5 during chondrogenic differentiation. A scrambled siRNA duplex purchased from Eurogentec was used as control condition for both *Sox9* or *Bapx1* knockdown. In addition, ATDC5 cells transfected with the scrambled siRNA were exposed to 30 ng/ml BMP-2 from the start of differentiation (cells from the BMP-2 condition were also transfected with the negative control scrambled siRNA in order to make all three interventions (*Sox9* knockdown, *Bapx1* knockdown and exposure to BMP-2) technically comparable to the same control). RNA was isolated from these cultures at day 10 in differentiation. Gene expression of *Sox9* (**A**), *Bapx1* (**B**), *Col2a1* (**C**), *Aggrecan* (**D**), *Runx2* (**E**), *Mef2c* (**F**), *Col10a1* (**G**) and *Rmrp* RNA (**H**) was determined by RT-qPCR. Gene expression is depicted as fold induction relative to t = 0. Data was normalized to *β-actin* expression and represents the average values of 3 biological replicates plus standard deviation. For statistical evaluation an independent samples t-test was performed relative to control using Graphpad Prism 5. p-values are indicated. Experiment was performed three times with presented graphs being representative examples.

**Figure 4:**
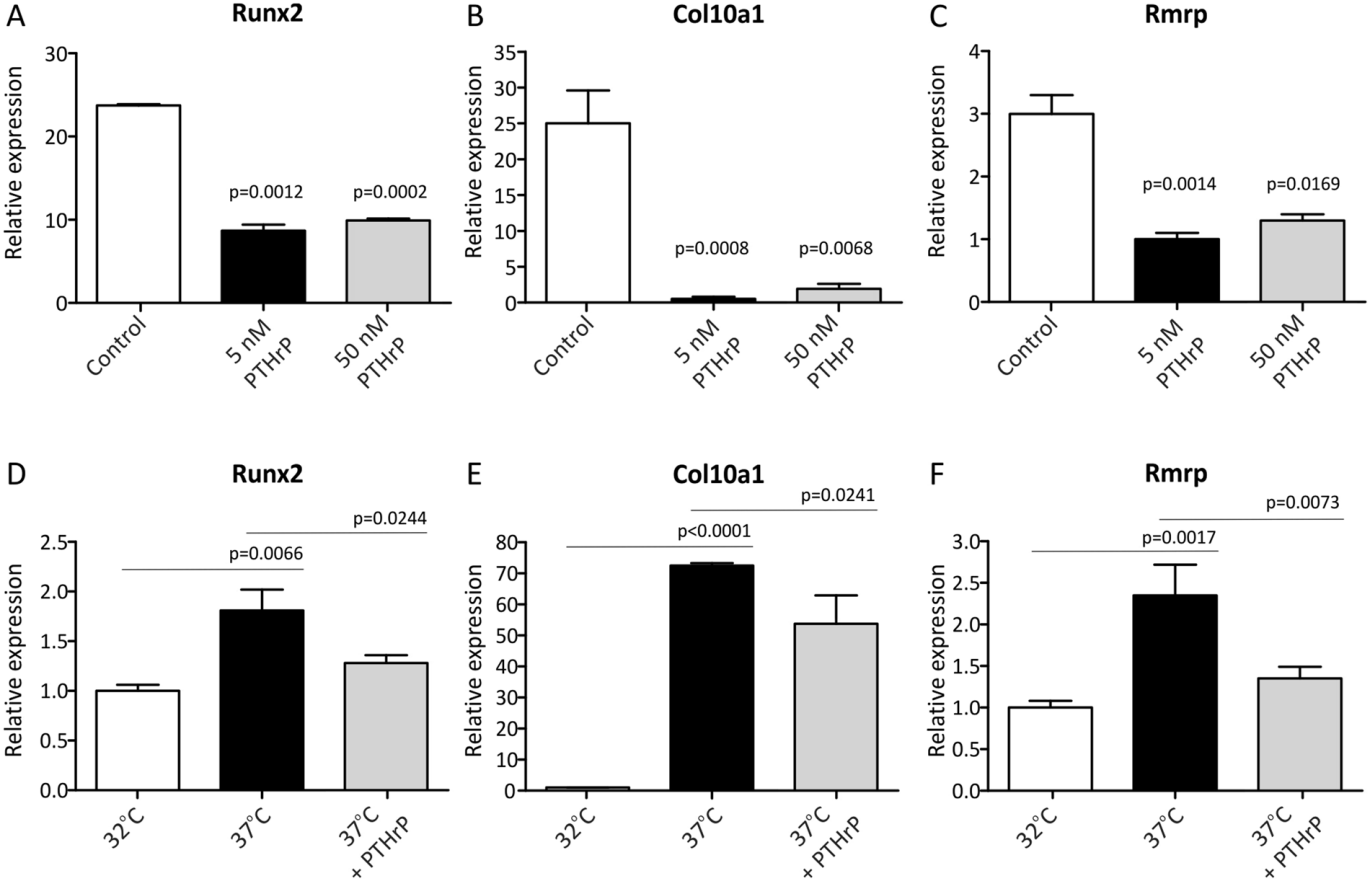
Hypertrophy-associated *Rmrp* RNA expression is reduced by PTHrP. ATDC5 cells were differentiated for 14 days to create a hypertrophic phenotype and 5 or 50 nM PTHrP was added from day 10 in chondrogenic differentiation onwards. Gene expression of *Runx2* (**A**) and *Col10a1* (**B**) and *Rmrp* RNA (**C**) was analyzed by RT-qPCR. Data was normalized to *β-actin*, represents the average of value of 3 individual samples plus standard deviation and is depicted as fold induction relative to day 0 ATDC5 cells. For statistical evaluation an independent samples t-test was performed relative to day 14 control ATDC5 cells using Graphpad Prism, where *= p < 0.05. MCT cells were plated and cultured at the permissive temperature of 32 °C and hypertrophic differentiation was induced by incubating the cells at 37 °C in the absence or presence of 50 nM PTHrP. Total RNA was isolated 24 hours after the temperature shift and analyzed for *Runx2* (**D**), *Col10a1* (**E**) and *Rmrp* RNA (**F**) expression by RT-qPCR. Data was normalized to *β-actin*, represents the average value of 4 biological replicates plus standard deviation and is depicted as fold-induction relative to the permissive temperature of 32 °C. For statistical evaluation an independent samples t-test was performed between the permissive temperature and induction of hypertrophic differentiation and between hypertrophic induction in the absence or presence of PTHrP using Graphpad Prism 5. p-values are indicated. Presented graphs are representative examples of three independent experiments.

Above data indicate that *RMRP* RNA expression is associated with late phase/hypertrophic chondrogenic differentiation. Therefore we next asked whether *RMRP* RNA expression adapts to a changing chondrocyte hypertrophic phenotype. To drive differentiating ATDC5 cells towards a hypertrophic phenotype we reduced the expression of Sox9 or Bapx1 by targeting the corresponding mRNAs by siRNA-mediated knockdown. In line with previous reports and work from our group^33, 35–37^, knockdown of *Sox9* expression (Fig. 3A) resulted in reduced expression of chondrogenic markers *Bapx1* (Fig. 3B), *Col2a1* (Fig. 3C) and *Aggrecan* (Fig. 3D) and concomitantly increased expression of hypertrophic markers *Runx2* (Fig. 3E), *Mef2c* (Fig. 3F) and *Col10a1* (Fig. 3G), which is consistent with a prominent hypertrophic chondrocyte phenotype. As a result of *Sox9* knockdown, the expression of *Rmrp* RNA (Fig. 3H) was increased. We previously reported^35^ that knockdown of the key hypertrophic repressor *Bapx1* (Fig. 3B) does not influence the expression of chondrogenic factors *Sox9*, *Col2a1* and *Aggrecan* (Fig. 3A,C,D). Instead, a selective hypertrophic chondrocyte phenotype was provoked, as evidenced by increased expression of hypertrophic markers *Runx2*, *Mef2c* and *Col10a1* (Fig. 3E–G). Similar to what was observed for *Sox9* knockdown, preferential hypertrophic differentiation by knockdown of *Bapx1* expression led to increased expression of *Rmrp* RNA (Fig. 3H). Increased hypertrophy was also observed when ATDC5 cells were exposed to the hypertrophic inducer BMP-2^36^, as evidenced by induced expression of hypertrophic markers. Under these conditions, the expression of *Rmrp* RNA was also induced (Fig. 3H). To further investigate *Rmrp* induction by chondrocyte hypertrophy, ATDC5 cell were differentiated for 14 days to induce a hypertrophic phenotype. To counteract hypertrophy, cultures were exposed to PTHrP from day 10 in differentiation onwards^23, 38^. Addition of PTHrP reduced the induction of the hypertrophic markers *Runx2* (Fig. 4A) and *Col10a1* (Fig. 4B). As a result of PTHrP-mediated reduction of hypertrophy, *Rmrp* RNA expression was downregulated as well (Fig. 4C). To confirm the PTHrP-mediated reduction of *Rmrp* RNA expression in another hypertrophic chondrocyte model, we used MCT cells. MCT cells are mouse chondrocytes, immortalized with a temperature-sensitive simian virus 40 large tumor antigen. These cells proliferate at 32 °C, but terminally differentiate and become hypertrophic within 24 hours at 37 °C^32^. As expected, culturing MCT cells at 37 °C induced hypertrophic differentiation as indicated by upregulation of *Runx2* and *Col10a1* expression (Fig. 4D,E). *Rmrp* RNA expression was also upregulated in this hypertrophic cell model (Fig. 4F). When the hypertrophic MCT phenotype was suppressed by PTHrP (Fig. 4D,E), *Rmrp* RNA expression responded similarly (Fig. 4F). Overall, our data indicate a positive correlation between *Rmrp* RNA expression levels and chondrocyte hypertrophy.

### Chondrogenic morphogens alter Rmrp promoter activity

**Figure 5:**
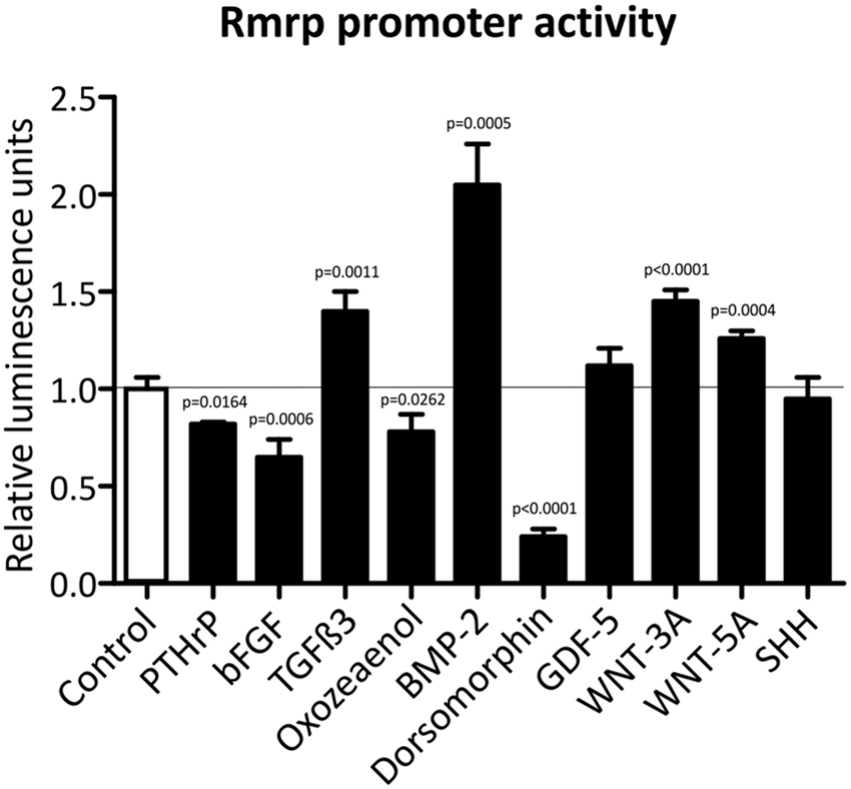
*Rmrp* promoter activity is altered by chondrogenic morphogens. *Rmrp* promoter responsiveness to different chondrogenic morphogens and pathways was determined by transfection of the pGluc-Rmrp-prom reporter plasmid, comprising the 1500 nucleotide sequence upstream of the mouse *Rmrp* RNA transcription start site. ATDC5 cells were transiently co-transfected with this reporter and pGL4.20[luc2/Puro]-CMV to normalize for transfection efficiency. Transfected cells were exposed for 24 hours to either 100 nM PTHrP, 15 ng/ml bFGF, 10 ng/ml TGFβ3, 0.5 μM (5*Z*)-7-Oxozeaenol, 30 ng/ml BMP-2, 10 μM dorsomorphin, 100 ng/ml GDF-5, 15 ng/ml WNT-3A, 15 ng/ml WNT-5A or 150 ng/ml SHH. These concentrations were chosen based on their use as described in literature. Promoter activity is depicted as fold induction relative to control. Data was normalized to pGL4.20[luc2/Puro]-CMV and represents the average value of 4 biological replicates plus standard deviation. For statistical evaluation an independent samples t-test was performed relative to control using GraphPad Prism 5. p-values are indicated. Presented graphs are representative examples of three independent experiments.

Our data described above suggested that *RMRP* RNA expression may be responsive to common chondrogenic morphogens, for example via modulation of proximal promoter activity. To acquire a broader understanding of chondrogenic pathways that may influence *RMRP* RNA expression, ATDC5 cells were transfected with a Gaussia luciferase reporter, driven by the 1500 nucleotide sequence upstream of the mouse *Rmrp* transcription start site (pGluc-Rmrp-prom plasmid) and cells were exposed to different chondrogenic mediators (Fig. 5). PTHrP keeps chondrocytes in a proliferative state and counteracts hypertrophic differentiation^23, 38^. PTHrP decreased *Rmrp* promoter activity by 18%. bFGF (or FGF2), a negative regulator of chondrocyte hypertrophic maturation^39^, reduced *Rmrp* promoter activity by 35%. TGFβ isoforms activate SMAD signaling via SMAD2/3^40^. Of all TGFβ isoforms, TGFβ3 was described to be able to induce matrix mineralization^41^. Indeed, TGFβ3 increased *Rmrp* promoter activity by 40%. In agreement with the TGFβ3-induced increase of *Rmrp* promoter activity, exposure to (5*Z*)-7-Oxozeaenol, an inhibitor of TGF-β activated kinase-1 (TAK1)^42^, decreased *Rmrp* promoter activity by 22%. Exposure to BMP-2 (pro-hypertrophic, see Fig. 3) increased *Rmrp* promoter activity by 105%, whereas dorsomorphin, an inhibitor of BMP-mediated SMAD1/5/8 phosphorylation^43^, decreased *Rmrp* promoter activity by 76%. GDF-5 (a BMP family member described to induce both chondrogenic differentiation and hypertrophy^44^ did not significantly change *Rmrp* promoter activity. WNT-3A and WNT-5A, two prominent ligands of the Wnt signaling pathway and crucial in chondrocyte differentiation^45^, increased *Rmrp* promoter activity by 45% and 26%, respectively. Finally, Sonic Hedge Hog (SHH), the main ligand that activates Patched-1-mediated GLI-signaling and which is crucial in limb bud patterning during embryogenesis^46^, did not significantly alter promoter activity. We thus concluded that the 1500 nucleotide sequence upstream of the *Rmrp* RNA transcription start site is responsive to mediators known to alter the chondrocyte phenotype. This indicates that, in chondrocytic cells, *Rmrp* RNA levels may be controlled by transcription factors involved in chondrocyte differentiation and hypertrophy.

### Rmrp RNA knockdown deregulates chondrogenic differentiation of ATDC5 cells

**Figure 6:**
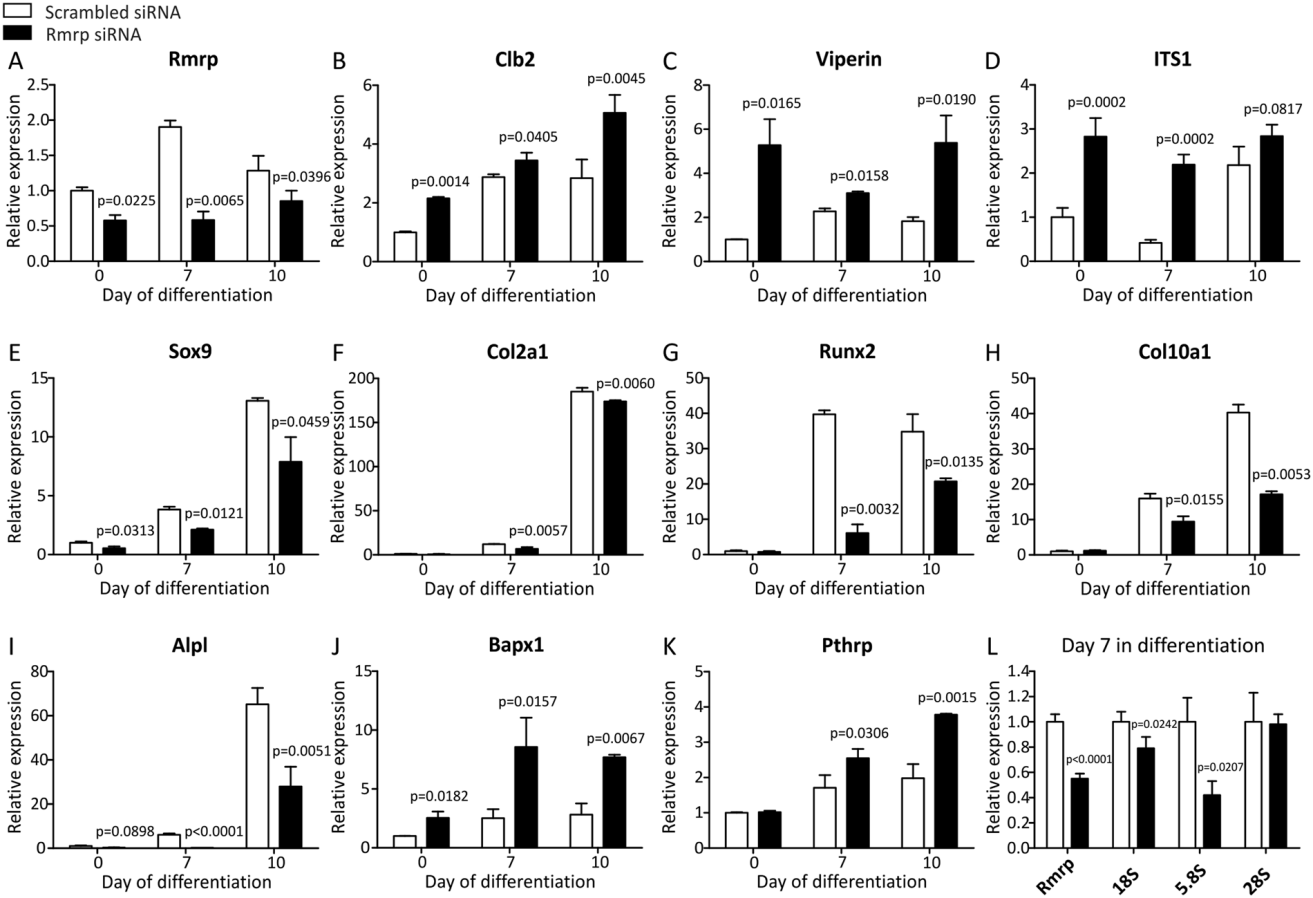
*Rmrp* RNA knockdown deregulates ATDC5 chondrogenic differentiation. ATDC5 cells were transfected with 100 nM siRNA duplex targeting *Rmrp* RNA expression on day -1, day 2, and day 5 during chondrogenic differentiation. A scrambled siRNA was used as control condition. RNA was isolated from these cultures at day 7 and 10 in differentiation and at t = 0 as a reference. Expression of *Rmrp* (**A**), *Clb2* (**B**), *Viperin* (**C**), ITS1 rRNA processing intermediate (**D**), *Sox9* (**E**), *Col2a1* (**F**), *Runx2* (**G**), *Col10a1* (**H**), *Alpl* (**I**), *Bapx1* (**J**), *PthrP* (**K**) and *Rmrp*, 18S, 5.8S and 28S (**L**) was determined by means of RT-qPCR. Gene expression is depicted as fold induction relative to t = 0 (**A–K**). In Fig. 6L day 7 scrambled controls are set to 1. Data was normalized to *β-actin* and represents the average value of 3 (Fig. 6A–K) or 4 (Fig. 6L) biological replicates plus standard deviation. For statistical evaluation an independent samples t-test was performed relative to scrambled control for each consecutive time point using GraphPad Prism 5. p-values are indicated. Presented graphs are representative examples of three independent experiments.

To investigate whether *RMRP* RNA regulates chondrogenic differentiation, *Rmrp* RNA expression was targeted by RNAi. ATDC5 cells were transfected with an siRNA duplex at day -1, 2 and 5 during chondrogenic differentiation and gene expression was determined at day 0, 7 and 10 in differentiation. The siRNA duplex reduced the expression of *Rmrp* RNA significantly (Fig. 6A). To confirm that this led to a functional reduction of RNase MRP activity, the expression of substrate RNAs for RNase MRP, i.e. *Clb2* mRNA, *Viperin* mRNA and ITS1 pre-rRNA, were measured^4–6^. Reduced *Rmrp* RNA levels indeed resulted in elevated levels of RNase MRP substrates *Clb2* and *Viperin* mRNAs, as well as an accumulation of an ITS1 pre-rRNA processing intermediate. (Fig. 6B–D). Following *Rmrp* knock-down, expression of *Sox9* (Fig. 6E), *Col2a1* (Fig. 6F), *Runx2* (Fig. 6G), *Col10a1* (Fig. 6H) and *Alpl* (Fig. 6I) was reduced at both 7 and 10 days in differentiation. Overall, expression of *Runx2*, *Col10a1* and *Alpl* seemed to be more heavily affected than *Col2a1*. In accordance, *Bapx1* mRNA was strongly induced (Fig. 6J), which is indicative of deregulated hypertrophic differentiation^35^. These data show that reduction of *Rmrp* RNA levels impacts the course of chondrogenic differentiation, with a prominent impact on the hypertrophic differentiation program. In concordance with the observed deregulated chondrogenic differentiation, expression of *Pthrp* was increased after *Rmrp* RNA knockdown (Fig. 6K). Since RNase MRP has been shown to be implicated in pre-rRNA processing^3, 4^, we assessed the levels of 18 S, 5.8 S and 28 S following *Rmrp* RNA knockdown and we observed reduced levels of 18 S and 5.8 S, but not of 28 S rRNAs at day 7 in ATDC5 chondrogenic differentiation (Fig. 6L).

### Rmrp RNA knockdown affects cell proliferation, glycosaminoglycan content and mineralization of ATDC5 cells

**Figure 7:**
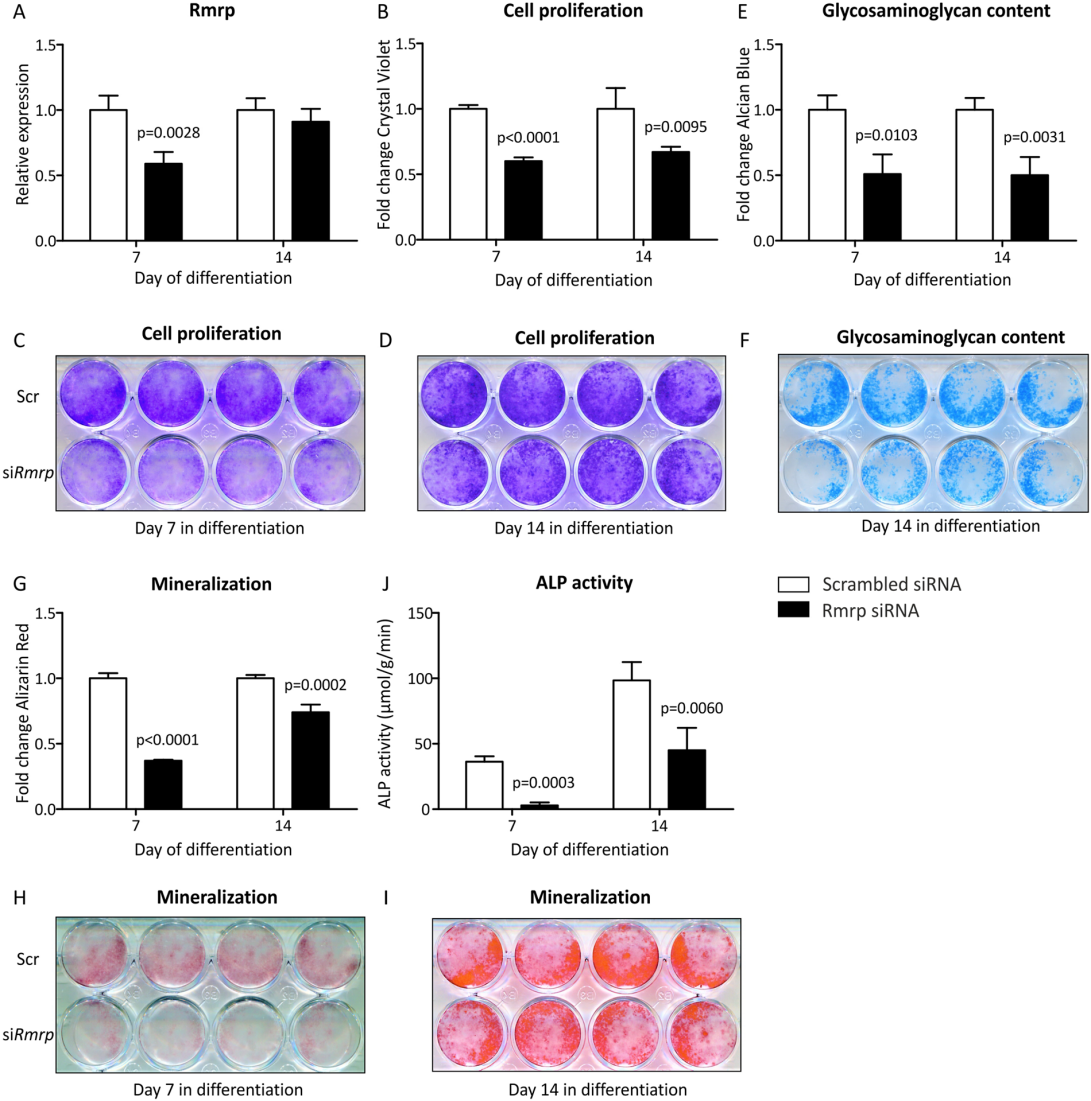
*Rmrp* RNA knockdown reduces cell proliferation, glycosaminoglycan content and mineralization. ATDC5 cells were transfected with siRNA duplex targeting *Rmrp* RNA on day 2 and 5 in differentiation (30 and 100 nM, respectively) and analyzed on day 7. In addition differentiating ATDC5 cells were transfected with the *Rmrp* RNA siRNA at day 2, 5 and 8 (30 nM, 100 nM and 100 nM, respectively) and analyzed at day 14 in differentiation. A scrambled siRNA was used as a control. Knockdown of *Rmrp* RNA expression was confirmed by RT-qPCR (**A**). Cell proliferation was determined by extraction and spectrophotometric analysis of bound Crystal Violet staining (**B**); staining before dye extraction is shown in (**C**) and (**D**). Glycosaminoglycan content was determined by extraction of Alcian Blue staining (**E**); day 14 staining before dye extraction is shown in (**F**). Mineralization was determined by extraction of Alizarin Red staining (**G**); staining before dye extraction is visualized in (**H**) and (**I**). ALP activity in μmol/g/min, corrected for total protein was determined in (**J**). Data (**A,B,E,G,J**) represents the average value of 4 biological replicates plus standard deviation and scrambled control is set to 1 (**A,B,E,G**). In graph (**J**) ALP activity in μmol/g/min was corrected for total protein levels. For statistical evaluation (**A,B,E,G,J**) an independent samples t-test was performed relative to scrambled control for each consecutive time point using GraphPad Prism 5. p-values are indicated. In (**C,D,F,H,I**) the upper panels show 4 biological replicates ‘Scr’ = Scrambled siRNA and the lower panels show 4 biological replicates ‘si*Rmrp*’ = *Rmrp* siRNA.

We next investigated whether deregulation of chondrogenic differentiation by *Rmrp* RNA knockdown is accompanied by functional changes of the chondrogenic differentiation program at different moments in differentiation. To maintain *Rmrp* knockdown during an extended period of time during chondrogenic differentiation, siRNAs were either transfected on day 2 and 5 in chondrogenic differentiation for read-out at day 7 or on day 2, 5 and 8 in chondrogenic differentiation for read-out at day 14. *Rmrp* RNA knockdown resulted in reduced *Rmrp* RNA expression at day 7 in chondrogenic differentiation (Fig. 7A). Since day 14 cells were last transfected at day 8 in chondrogenic differentiation, *Rmrp* RNA expression was almost back to baseline levels in the day 14 *Rmrp* RNA knockdown condition (Fig. 7A). *Rmrp* RNA knockdown resulted in reduced cell numbers, inferring reduced proliferation (quantified in Fig. 7B and visually depicted in Fig. 7C,D). *Rmrp* RNA knockdown led to a significant reduction of GAG content at day 7 and this was still detectable at day 14 in chondrogenic differentiation (Fig. 7E). GAG content at day 14 before extraction of the Alcian Blue dye is visualized in Fig. 7F. Mineralization, quantified by Alizarin Red staining was reduced at day 7 and day 14 in chondrogenic differentiation (Fig. 7G) and visualized in Fig. 7H,I. In concert with the Alizarin Red data, ALP enzyme activity was almost absent at day 7 in chondrogenic differentiation as a result of *Rmrp* knockdown and was still significantly reduced at day 14 in chondrogenic differentiation (Fig. 7J).

### Chondrogenic trans-differentiation is impaired in CHH fibroblasts

**Figure 8:**
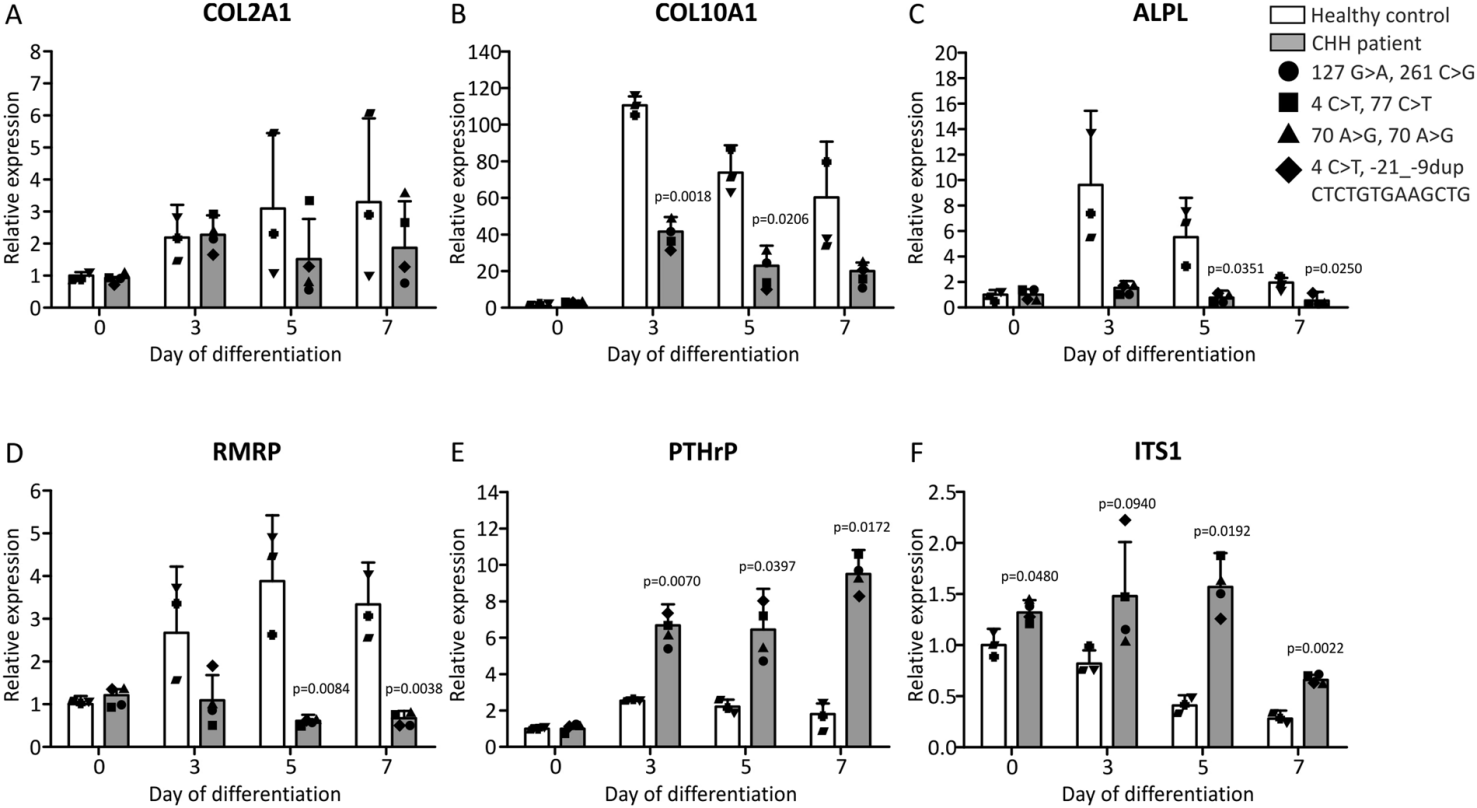
Impaired chondrogenic differentiation and increased ITS1 expression during chondrogenic trans-differentiation of CHH fibroblasts. Human dermal fibroblasts from four CHH patients (RMRP alleles of CHH patients carried the following mutations: 127 G > A and 261 C > G (circle); 4 C > T and 77 C > T (square); 70 A > G and 70 A > G (triangle); 4 C > T and -21_-9dup CTCTGTGAAGCTG (diamond)) and three healthy controls (individual gene expression data points for each healthy control and patient are indicated) were trans-differentiated into the chondrogenic lineage by hyperconfluent plating in wells coated with Aggrecan. Total RNA was isolated at days 0 (non-plated fibroblasts), 3, 5 and 7 in trans-differentiation and gene expression of *COL2A1* (**A**), *COL10A1* (**B**), *ALPL* (**C**), *RMRP* (**D**), *PTHrP* (**E**) and ITS1 (**F**) was determined by RT-qPCR. Data was normalized to *GAPDH* and represents the mean plus standard deviation; gene expression data is set relative to day 0 healthy control. For statistical evaluation an independent samples t-test was performed relative to healthy control for each individual time point using GraphPad Prism 5. p-values are indicated. Graphs are representative examples of three independent experiments.

CHH is caused by mutations in *RMRP* RNA^14, 17^, but it is unknown whether these mutations influence chondrogenic differentiation in CHH patients. To test whether the chondrogenic capacity of CHH cells is impaired, we employed a trans-differentiation protocol that drives dermal fibroblasts towards a chondrocyte-like phenotype^34^. High density plating of fibroblasts on an Aggrecan-coated surface in the presence of TGFβ3 induced the formation of dense aggregates within 24 hours post-plating, resembling chondrogenic nodules. To focus on functional chondrogenic read-out markers we measured induction of *COL10A1*, *COL2A1* and *ALPL* (alkaline phosphatase) gene expression in cultures from healthy fibroblasts, substantiating trans-differentiation into the chondrogenic lineage (Fig. 8A–C). Induction of *COL10A1* expression was more robust than *COL2A1*, which indicates that these cultures display a predominant hypertrophic phenotype (Fig. 8A,B). As a result of the chondrogenic trans-differentiation *RMRP* RNA expression levels increased (Fig. 8D). Compared to healthy control cultures we found that CHH fibroblasts (4 CHH patients, carrying different CHH pathogenic mutations in the RMRP gene (i.e. 127 G > A and 261 C > G; 4 C > T and 77 C > T; 70 A > G and 70 A > G; 4 C > T and -21_-9dup CTCTGTGAAGCTG)) displayed an impaired induction of *COL10A1* and *ALPL* expression upon chondrogenic trans-differentiation (Fig. 8B,C). Induction of *COL2A1* expression was not affected at day 3 in trans-differentiation. At days 5 and 7 during chondrogenic trans-differentiation, *COL2A1* expression appeared lower in CHH fibroblasts as compared to healthy controls (Fig. 8A), although this difference was not significant. The induction of *RMRP* RNA expression observed in healthy control fibroblasts was absent in CHH cultures (Fig. 8D). Moreover, and in concert with the observed impaired hypertrophic differentiation (Fig. 8B,C), the expression of PTHrP was increased in CHH cultures as compared to healthy control cultures (Fig. 8E). Finally, we detected a significant increase in the accumulation of the ITS1 pre-rRNA processing intermediate^4^ (Fig. 8F). Together, data show that in CHH fibroblasts chondrogenic trans-differentiation is impaired with a major impact on hypertrophic development of these cultures.

## Discussion

The involvement of the *RMRP* lncRNA in the pathophysiology of CHH has previously been identified^17^; however it is not clear why mutations in *RMRP* RNA lead to a severe skeletal dysplasia phenotype. Skeletal development depends on chondrogenic differentiation in the growth plate and therefore we hypothesized that *RMRP* RNA has a functional role during chondrogenic differentiation, explaining the dwarfism that characterizes CHH patients. We found that expression levels of *RMRP* RNA and RNase MRP protein subunits are induced and spatiotemporally regulated during the course of chondrogenic differentiation. *RMRP* expression levels are especially induced during the hypertrophic phase of chondrogenic differentiation, indicating an increased demand for *RMRP* RNA levels during this phase of the differentiation process. As long as the substrate(s) of RNase MRP that are involved in the molecular mechanisms of chondrogenic differentiation has/have not been identified, we can only speculate how induction of *RMRP* RNA expression is associated with chondrocyte hypertrophy. It is remarkable that in CHH tissues with fast-dividing cell types (growth plate, hair follicles, bone marrow etc.) seem to be the most affected ones^16^, suggesting a role for *RMRP* RNA in cell cycle regulation. Tight control over cell mitosis and differentiation is paramount to controlling growth plate development^47^. The proliferative zone of the growth plate presents a high mitotic activity, while terminally differentiated chondrocytes in the hypertrophic zone most likely lack a mitotic cycle. One of the identified activities of the RNase MRP complex is endoribonucleolytic cleavage of the mRNA of cyclin b2 (*Clb2*). This B-type cyclin activates cyclin-dependent kinase-1 (CDK1) during M-phase and anaphase-promoting complex (APC)-dependent proteolysis of Clb2 is essential for mitotic exit^48^. Low Clb2 levels also keep CDK1 activity to a minimum during G0 phase via similar APC-dependent proteolysis^49^. It is likely that the strictly regulated terminal differentiation of hypertrophic chondrocytes is orchestrated by mitotic exit or entrance into G0 phase. As RNase MRP activity has been shown to degrade the Clb2 message it is tempting to speculate that the elevation of RNase MRP levels during chondrocyte hypertrophy serves to decrease Clb2 protein levels by increased turnover of Clb2 mRNA to inhibit activation of CDK1. Compelling evidence from recent work indeed shows the crucial involvement of CDK1 in controlling chondrocyte hypertrophic differentiation^50^. CDK1 expression was found to be highly expressed in proliferative chondrocytes and was greatly diminished in hypertrophic chondrocytes in differentiating ATDC5 cultures, as well as in mouse growth plates. In addition genetic interference with CDK1 expression caused absence of proliferative chondrocytes in the growth plate and a switch towards hypertrophic differentiation in ATDC5 cultures, strongly suggesting that the reduction of CDK1 levels or its decreased activity is a prerequisite for hypertrophic differentiation. We thus speculate that in a healthy growth plate the increased RMRP RNA levels in hypertrophic chondrocytes may lead to decreased Clb2 levels, thereby hampering CDK1 activity and inducing an overall mitotic arrest. Work from the same group^50^ shows that the proliferation-promoting and hypertrophy-suppressive action of PTHrP is, at least in part, CDK1-dependent. In agreement with this notion we observed that *Rmrp* RNA levels are down regulated upon PTHrP exposure, presumably via decreased *Rmrp* promoter transcriptional activity. This may result in elevated Clb2 levels, thereby potentially aiding in PTHrP downstream CDK1 activity. In this respect, it is important to note that knockdown of *Rmrp* RNA levels during ATDC5 chondrogenic differentiation increases Clb2 and *Pthrp* mRNA levels and deregulates chondrogenic differentiation in an overall hypertrophy-suppressing fashion. One of the earliest identified roles of RNase MRP is endoribonucleolytic maturation of 5.8 S rRNA by cleaving site A3 in the internal transcribed spacer 1 (ITS1) in yeast^3, 4^. Even though there is presently no conclusive evidence showing that specifically hypertrophic chondrocytes in the growth plate display the highest protein synthetic capacity, an RMRP RNA/RNase MRP mediated contribution to the synthesis of the large ribosomal subunit in the growth plate may support the high protein synthetic activity of the growth plate to produce protein-rich cartilaginous extracellular matrix. For long an involvement of RNase MRP in the processing of ITS1 in human cells remained elusive^51^. However, recently it has been elegantly shown that human RMRP RNA in the RNase MRP complex indeed catalyzes the endoribonucleolytic cleavage of ITS1, thereby contributing to pre-rRNA maturation^4^. Indeed our RMRP RNA knockdown data in ATDC5 show accumulation of an ITS1 pre-rRNA processing intermediate as well as reduced levels of mature 5.8 S and 18 S rRNA. In concert with these findings we detected similar accumulation of an ITS1 processing intermediate in differentiating CHH cells. Chondrogenic differentiation of ATDC5 and the developing growth plate is associated with high proliferative capacity^31^ and increased synthesis of protein-rich ECM. It is conceivable that this alters the cellular demand for mature rRNAs for ribosome biogenesis, potentially explaining the changes in *Rmrp* RNA expression during chondrogenic differentiation and the phenotype observed after *Rmrp* RNA knockdown. Finally, *RMRP* RNA has been described to associate with TERT^7^, the reverse transcriptase that is associated with the telomerase holoenzyme. This *RMRP* RNA-containing macromolecular complex was demonstrated to display RNA-dependent RNA polymerase activity. This activity generates a double-stranded *RMRP* RNA molecule that is converted by Dicer into an siRNA that targets *RMRP* RNA^7^, as well as specific mRNAs^52^. Other *RMRP* RNA-derived siRNAs have been identified as well, but it is currently unknown whether these are also generated via a similar mechanism. The latter siRNAs, termed *RMRP*-S1 and *RMRP*-S2^8^, seem to target genes relevant for skeletal development like *SOX4*, *PTCH2* and *BMPR2*. It remains elusive whether this relates to increased *RMRP* RNA levels in hypertrophic chondrocytes, but it is conceivable that upregulation of *RMRP* RNA leads to higher production of *RMRP*-S1 and *RMRP*-S2, which in turn may change the magnitude by which *RMRP*-S1 and *RMRP*-S2 targets are influenced.

Our observations that the expression of RNase MRP components is modulated in differentiating cells promoted us to investigate whether *RMRP* RNA expression levels can be controlled at the transcriptional level. *RMRP* RNA expression is driven by RNA polymerase III (RNAPIII) and the proximal *RMRP* RNA promoter has been studied in the past to some extent^53^, showing that the −84 bp promoter region is sufficient to drive RNAPIII-dependent transcription of *RMRP* RNA. However, to fine-tune promoter activity and to be able to respond to alternating demands of *RMRP* RNA (e.g. during chondrocyte hypertrophy), it is expected that additional transcription regulatory elements are present. Indeed, WNT-3A was recently found to be able to drive *RMRP* transcription in cancer via activation of β-catenin and YAP proteins^54^. Here, we showed that the 1500 base pair sequence upstream of the *Rmrp* RNA transcription start site is responsive to a series of chondrocyte morphogens or pathways. Several of the growth factors tested increased or decreased the transcriptional activity of the minus 1500 *Rmrp* promoter, indeed indicating that in chondrocytic cells transcription of *Rmrp* RNA may be under transcriptional control of chondrogenic cues. In agreement with their known role in chondrocyte hypertrophy^45^, WNT-5A, WNT-3A and BMP-2 induced *Rmrp* promoter activity, suggesting that increased *Rmrp* RNA expression in chondrocyte hypertrophy is, at least in part, transcriptionally controlled by one or more of these morphogens and their downstream pathways. Indeed, *in silico* prediction of putative transcription factor binding sites in the minus 1500 bp *Rmrp* promoter sequence suggests the presence of CREB, MEF2, TCF/LEF and SMAD binding sites (Genomatix; data not shown) which are transcription factors acting downstream of WNT-5A, WNT-3A and BMP-2 signaling. Dorsomorphin (a BMP/SMAD1/5/8 inhibitor) exposure greatly reduced *Rmrp* promoter activity, further supporting BMP-mediated control of *RMRP* transcription. In keeping with a potential feedback mechanism it is noteworthy that BMPR2 (the type II BMP receptor) is a potential target of *RMRP*-S2, the *RMRP* RNA-derived siRNA^8^.

Active control over *Rmrp* transcription in chondrocyte hypertrophy is further substantiated by the observation that PTHrP and bFGF are able to reduce *Rmrp* promoter activity. PTHrP and bFGF delay chondrocyte hypertrophy/terminal differentiation^23, 38, 39, 55^. At this point we can only speculate whether reduced *Rmrp* promoter activity and *Rmrp* expression by PTHrP and bFGF is an indirect result of a morphogen-dependent changing chondrocyte hypertrophic phenotype (e.g. decreased WNT or BMP signaling), or that these morphogens directly control *Rmrp* abundance by downstream inhibition of *Rmrp* promoter activity. We conclude that *Rmrp* transcription in chondrocytes is controlled by well-known chondrogenic signaling pathways that are associated with hypertrophic differentiation. It remains to be determined whether such transcriptional control is also present in other cell types and how this communicates with the RNAPIII transcription machinery^56^.

The clinical presentation of CHH is dominated by short stature, caused by impaired skeletal development. Our data are consistent with a pivotal role for *RMRP* RNA during chondrogenic differentiation and is especially linked to chondrocyte hypertrophic differentiation. Genetic interference with *Rmrp* RNA expression in ATDC5 cells and chondrogenic trans-differentiation of CHH fibroblasts indicates delayed chondrocyte hypertrophy. Growth plates of CHH patients are characterized by delayed ossification and the presence of very few hypertrophic chondrocytes^25^. Whether and how delayed chondrocyte hypertrophy may influence the development of the growth plate in CHH remains to be determined. Finally, considering our observations that the expression of PTHrP is induced during knockdown of *Rmrp* RNA and in chondrogenic differentiating CHH fibroblasts and its inhibiting action on *RMRP* promoter activity and *RMRP* RNA expression, it is interesting to realize that disrupted PTH/PTHrP signaling is deleterious for skeletal development by endochondral ossification^57^. Considering this it is tempting to speculate whether there are molecular connections between *RMRP* RNA and PTH/PTHrP in skeletal dysplasias like cartilage-hair hypoplasia, metaphyseal chondrodysplasia Jansen type (OMIM #156400)^58^ and Blomstrand type chondrodysplasia^59^, (OMIM #215045).

In conclusion, lncRNAs involved in mesenchymal cellular differentiation processes are scarcely known^60^ and we found that the *RMRP* lncRNA is differentially expressed during different stages of chondrogenic differentiation and displaying a prominent association with chondrocyte hypertrophic development. Our findings shed new light on potential pathobiological mechanisms involved in the skeletal dysplasia phenotype associated with cartilage-hair hypoplasia.
